# Ligamentary Leiomyoma Presenting as a Mons Pubis Mass: A Rare Clinical Manifestation

**DOI:** 10.3390/diagnostics16203376

**Published:** 2026-10-17

**Authors:** Philip H. Klecker, Joscha Steetskamp, Annette Hasenburg, Yaman Degirmenci

**Affiliations:** Department of Obstetrics and Gynecology, University Medical Center of the Johannes Gutenberg University Mainz, Langenbeckstraße, 1, 55131 Mainz, Germany; josch.steetskamp@unimedizin-mainz.de (J.S.); annette.hasenburg@unimedizin-mainz.de (A.H.); yaman.degirmenci@unimedizin-mainz.de (Y.D.)

**Keywords:** ligamentary leiomyoma, inguinal canal, extrapelvic leiomyoma, Mons Pubis Mass

## Abstract

We present the case of a 47-year-old woman (gravida II, para II; both deliveries by cesarean section) with a rare case of a ligamentary leiomyoma appearing as a mass lateral of the mons pubis. The lesion had first been noticed approximately seven months prior to the first examination at our institution and had shown continuous growth since its initial detection. Apart from occasional mild discomfort, no additional symptoms such as skin changes, discharge, or systemic complaints were reported. Based on the clinical and sonographic findings, no suspicion of malignancy was raised during the preoperative assessment, and elective surgical excision was scheduled for both diagnostic and therapeutic purposes. The described lesion presented intraoperatively as a ligamentary leiomyoma and subsequent histopathological examinations confirmed the diagnosis.

**Figure 1:**
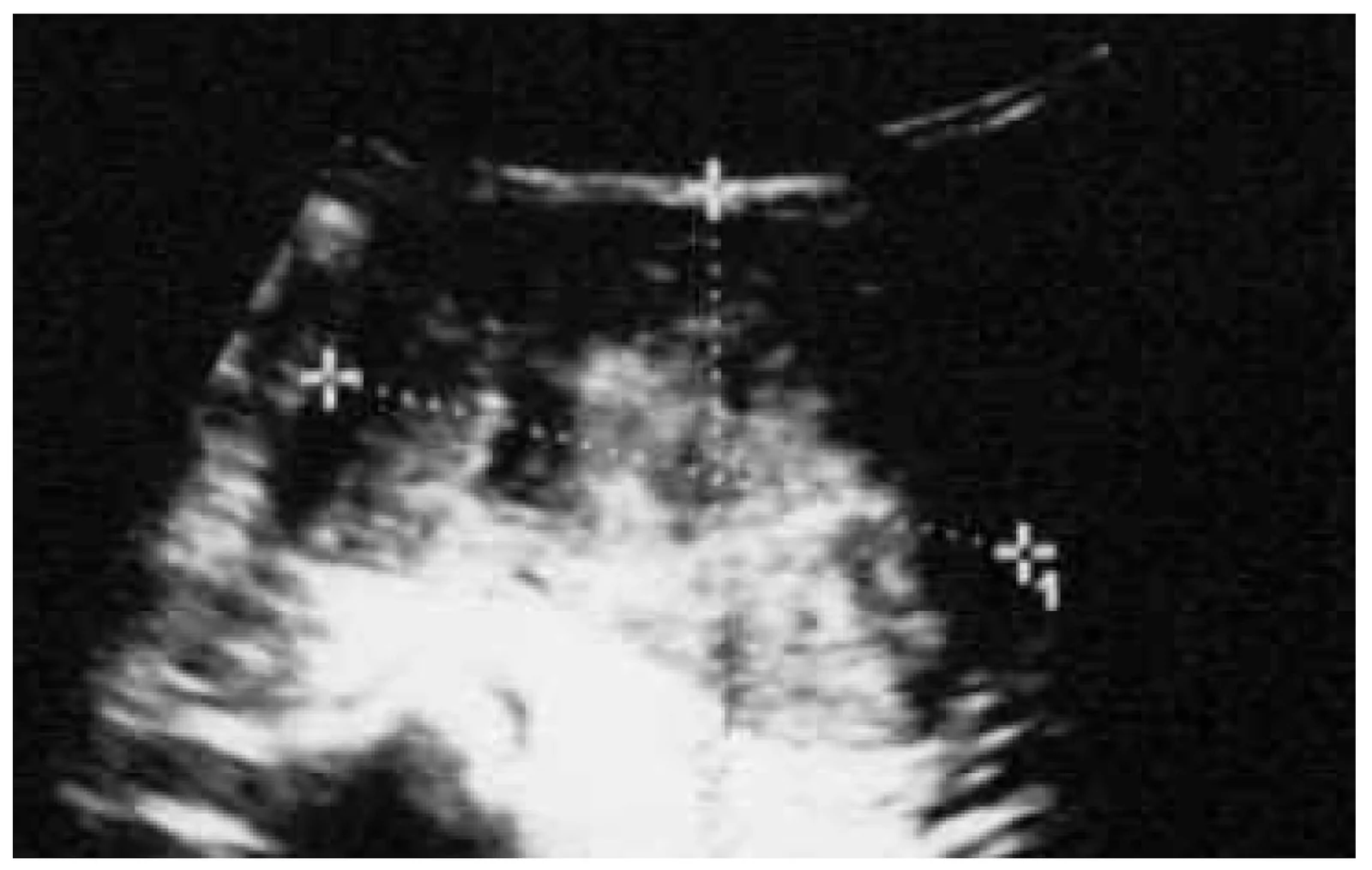
Clinical examination revealed a well-defined, freely mobile, non-tender soft tissue mass measuring approximately 6 × 4.5 cm limited to the region lateral of the left mons pubis without swelling of the groin region. Bilateral inguinal lymph nodes were neither enlarged nor indurated on palpation. Sonographic evaluation demonstrated a circumscribed, hypoechoic and heterogeneous lesion without evidence of increased vascularization on Doppler imaging. All visible lymph nodes appeared unremarkable on ultrasound examination. A vascularized pedicle in the region of the inguinal canal was not detected in the initial ultrasound examination. There was no suspicion of a malignancy and surgical removal was scheduled without further radiological imaging.

**Figure 2:**
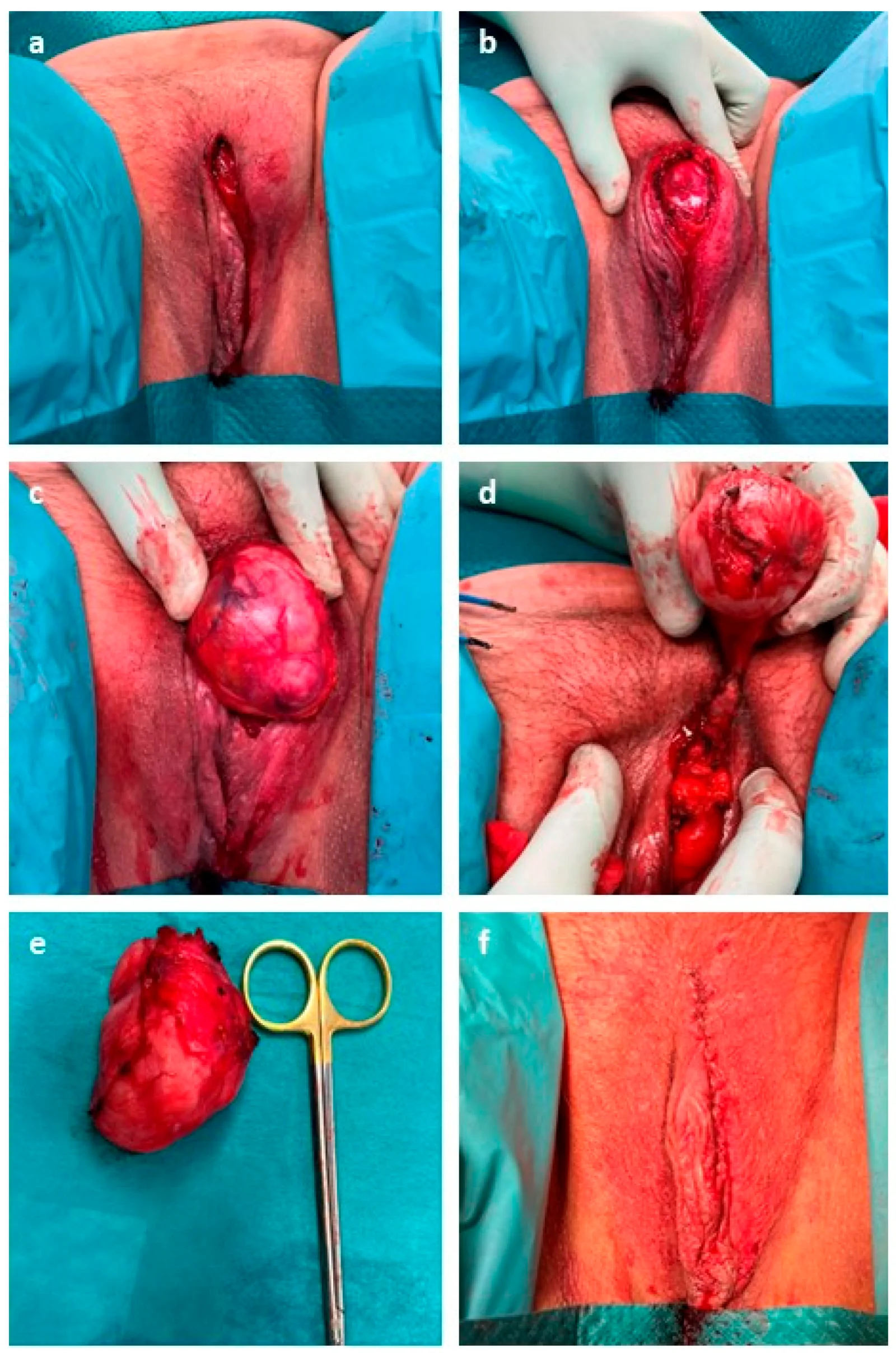
(**a**) Intraoperatively, a skin incision was tailored to the size and location of the lesion and placed along the natural skin tension lines while preserving the integrity of the labia majora. (**b**) Careful dissection of the surrounding tissues exposed (**c**) a well-circumscribed, solid tumor that was clinically suggestive of a leiomyoma. (**d**) Following further mobilization, a distinct vascularized pedicle was identified on the dorsal aspect of the mass. Gentle traction on this pedicle resulted in movement extending toward the course of the round ligament, raising the suspicion of a ligamentary leiomyoma that had developed in the inguinal and extended into the mons pubis region. However, due to the surgical approach, the exact anatomical origin of the lesion could not be identified. (**e**) After ligation and transection of the vascular pedicle, the tumor was completely excised. Macroscopically, the specimen appeared as a firm, well-circumscribed mass without obvious signs of infiltrative growth. (**f**) After excision of the lesion and individual hemostasis, the wound was closed with interrupted sutures, resulting in a good cosmetic outcome. The patient’s medical history was negative for inguinal hernia, uterine leiomyoma, or previous pathology involving the inguinal region. The postoperative course was uneventful, and the patient was discharged on the first postoperative day in good clinical condition and free of symptoms.

**Figure 3:**
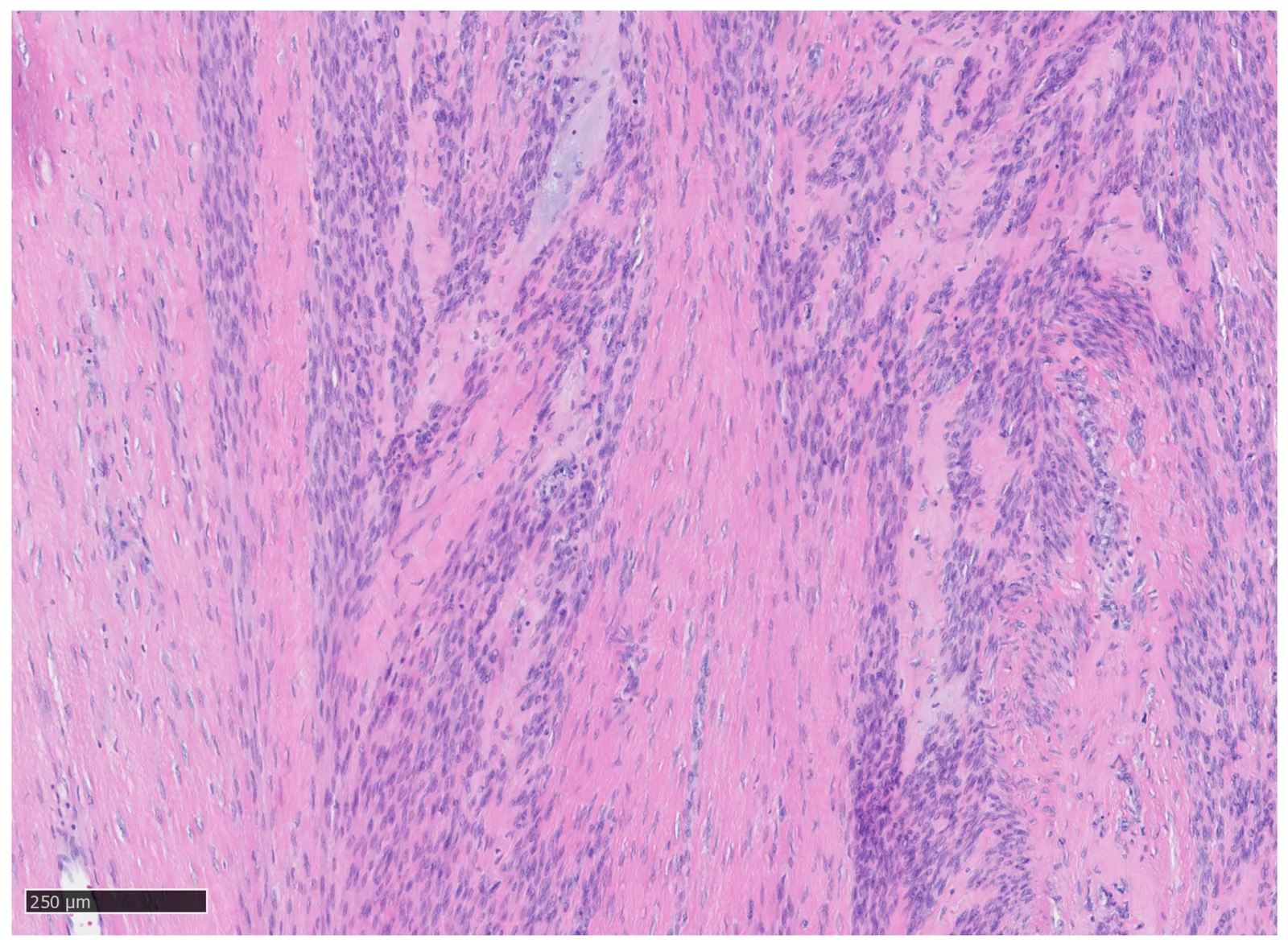
Macroscopically, the well-circumscribed tumor measured 6.8 × 5.0 × 4.5 cm. Histopathological evaluation showed a spindle-cell mesenchymal neoplasm consistent with a uterine-type leiomyoma. In addition to the HE staining, immunohistochemistry was performed and Smooth Muscle Actin (SMA), Caldesmon and Desmin were detected, confirming the clinical diagnosis. The uncommon location of the leiomyoma directly lateral of the mons pubis without an additional swelling in the groin region makes this case exceptionally rare. Our subsequent literature review showed only a few cases of ligamentary leiomyomas presenting as a mass in this particular position. In general, leiomyomas of the uterus are rather common and can appear in various locations mimicking other differential diagnoses such as ovarian [1] or inguinal masses [2] with a wide range of different sizes. Apart from originating directly from the uterus, leiomyomas can also be associated with the round ligament. In cases of round ligament leiomyomas, more than half present as extra-abdominal lesions, typically manifesting as a palpable inguinal mass. In approximately one-third of patients, a history of uterine leiomyomas has been identified as a potential predisposing factor. Other possible contributing factors are congenital abnormalities, the use of hormonal preparations [2], and pregnancy [3]. While most cases are thought to result from extra-abdominal or descending growth of a leiomyoma originating from the round ligament, one case reported a leiomyoma developing within the inguinal canal following laparoscopic hysterectomy. Implantation of myomatous cells following surgical resection can be a rare differential diagnosis [4]. The rarity of round ligament leiomyomas, combined with their various clinical presentations, often makes establishing a definitive diagnosis challenging. Important differential diagnoses include enlarged lymph nodes, inguinal hernias and hydroceles of the canal of Nuck [5]. In the reported cases of extra-abdominal round ligament leiomyomas, routine diagnostic evaluation primarily included abdominal and vaginal ultrasound, computed tomography (CT) and magnetic resonance imaging (MRI) [2,6]. However, in some cases surgical treatment was performed under the assumption of inguinal hernias, with the diagnosis of a leiomyoma being established incidentally during surgery [4,7]. Despite the diagnostic challenges, surgical excision is the treatment of choice for leiomyomas of the round ligament [2,8]. In conclusion, our case represents a rare manifestation of ligamentary leiomyoma appearing as a mass in the region of the mons pubis without additional swelling in the groin region. Therefore, ligamentary leiomyomas should be considered a rare differential diagnosis for masses in this region.

## Data Availability

The data presented in this study are available on request from the corresponding author. The data are not publicly available due to patient privacy.
